# Prevalence, Risk Factors and Cardiovascular Comorbidities of Resistant Hypertension among Treated Hypertensives in a Nigerian Population

**DOI:** 10.5334/gh.1296

**Published:** 2024-02-07

**Authors:** Olugbenga Olusola Abiodun, Tina Anya, Janefrances Chima Chukwu, Victor Adekanmbi

**Affiliations:** 1 Department of Internal Medicine, Federal Medical Centre, Abuja, Nigeria; 2 Trinity Health IHA Medical Group, 24 Frank Lloyd Wright Drive, Suite J2000 Ann Arbor, MI 48105, United States; 3 Department of Obstetrics and Gynaecology, University of Texas Medical Branch at Galveston, Texas, United States

**Keywords:** resistant hypertension, prevalence, cardiovascular comorbidities, Nigeria, African

## Abstract

The true prevalence and cardiovascular comorbidities of resistant hypertension (RH) in Nigeria and Africa are not known. We sought to determine the prevalence and cardiovascular comorbidities of resistant hypertension in a treated Nigerian hypertensive population.

We analyzed 1,378 patients with essential hypertension from a prospective clinical registry, the Federal Medical Centre Abuja Hypertension Registry. Resistant hypertension was defined as blood pressure ≥140/90 mmHg despite the use of ≥3 guideline-recommended antihypertensive medications including a diuretic, reninangiotensin system blocker and calcium-channel blocker at optimal or best-tolerated doses or blood pressure <140/90 mmHg on ≥4 antihypertensive medications. Resistant hypertension was confirmed with the use of home blood pressure monitoring while adherence was determined by monitoring prescription orders.

The prevalence of resistant hypertension was 15.5%, with 12.3% as controlled resistant hypertension and 3.3% as uncontrolled resistant hypertension. Risk factors independently associated with the odds of resistant hypertension were male sex (adjusted odds ratio [AOR]: 1.62, 95% confidence interval [CI] 1.19–2.21, p = 0.002), obesity, and diabetes mellitus. Furthermore, patients with resistant hypertension were more likely to have heart failure with preserved ejection fraction (AOR: 3.36, 95% CI 1.25–9.07, p = 0.017), cerebrovascular disease, and chronic kidney disease.

In our treated hypertensive cohort, resistant hypertension was associated with an increased risk of cerebrovascular disease, chronic kidney disease, and heart failure with preserved ejection fraction, and it appears this burden maybe 2–3 times more in those with resistant hypertension compared to those without. Concerted efforts to prevent or promptly treat resistant hypertension in our population will reduce cardiovascular comorbidities.

## Introduction

Resistant hypertension (RH) is a cause of considerable cardiovascular (CV) morbidity and mortality all over the world through cardiac, cerebrovascular, and renal diseases [1,2,3,4,5,6,7]. It is associated with increased financial burden due to polypharmacy, and the cost of care of co-morbidities or complications. The prevalence of true resistant hypertension is not accurately known globally. This is due to the evolving understanding of RH in the last two decades which occasioned the use of different definitions and methodologies. However, in the last decade and a half, consensus definitions have been more specific. In 2007 and 2008, the European and American guidelines defined RH as failure to achieve blood pressure (BP) control despite lifestyle modification and use of three or more antihypertensive medications in adequate doses, or four or more antihypertensive medications with or without BP control [8,9]. The specificity of present definitions was made possible by the increased understanding [10] of factors that cause falsely elevated office BP leading to pseudo-RH. These factors include inaccurate BP measurement, inadequate antihypertensive medication combinations, medication non-adherence, white coat hypertension, and treatment inertia [10]. The converse occurs when office BP is falsely controlled in the absence of out-of-office BP measurement that will show poor BP control. This is seen in patients with masked hypertension (MH), and this causes underestimation of RH. In cases where pseudo-resistance is excluded and MH is diagnosed with out-of-office BP measurement, true RH is said to be present.

In Nigeria and Africa, there is a dearth of information on the true prevalence, risk factors and CV comorbidities associated with RH. CV comorbidities such as cerebrovascular disease (CVD), heart failure (HF), chronic kidney disease (CKD) and coronary artery disease (CAD) in RH have been studied extensively in different populations but not in African populations [1,2,3,4,5,6,7]. In this study, we evaluated the prevalence, risk factors and CV comorbidities associated with RH in a cohort of treated hypertensive Nigerians. This will provide accurate and reliable information for policymaking and program design aimed at addressing the burden of resistant hypertension and related comorbidities in Nigeria and Africa.

## Materials and Methods

### Subjects

All consenting patients attending the cardiology clinics of Federal Medical Centre, Abuja (FMCA) were consecutively recruited into the Federal Medical Centre Abuja Hypertension Registry (FMCAHR) between 2016 and 2021. One thousand three hundred and seventy-eight (1,378) essential hypertensive patients aged 18 years and above were included in this study. All patients with secondary forms of hypertension including primary kidney diseases were excluded from this analysis.

The FMCA is a leading tertiary healthcare institution in the federal capital of Nigeria. Abuja has over one million ethnically diverse inhabitants. Also, nationals of other African nations and non-Africans domiciled in Abuja access health care in FMCA. All patients gave informed consent and ethics clearance with registration number FMCABJ/HREC/2017/009 was obtained from the hospital’s ethics research committee.

### Clinical information

Information was obtained from detailed medical history and examination and stored in an Excel database. These include age (years), sex, weight (kg), height (cm), body mass index (BMI) (kg/m^2^), waist circumference (cm), duration of hypertension and diabetes mellitus (DM) (in months and years), first-degree family history of hypertension and DM (parents, siblings and children), duration of alcohol use (grams and duration of use in years), tobacco use (pack years), office systolic and diastolic blood pressure (SBP & DBP) (mmHg) measurements by nurses and physicians at the first visit and the visit of BP control, number of clinic visits before BP control, home BP measurements and presence of CV comorbidities and non-CV comorbidities at recruitment, and on follow-up. Information was collected on all medications including combinations of antihypertensives.

### Laboratory evaluation

Blood and urine for urinalysis were collected at the hospital laboratory at the first clinic visit and on follow-up as appropriate for all routine investigations. The estimated glomerular filtration rate (eGFR) was calculated using the 2021 CKD epidemiology collaboration creatinine equation (CKD-EPI), and it was used to diagnose CKD when persistently < 60 mls/min/1.73 m^2^ [11].

A resting 12-lead electrocardiogram (ECG) was carried out on all participants on their first clinic visit and subsequently, as the need arose by trained technicians according to the American College of Cardiology guidelines [12]. Schiller AT-102 system (Baar, Switzerland) at a speed of 25 mm/s and 1 mV/cm calibration was used, and ECGs were reported by hospital cardiologists.

Echocardiography was performed on all patients by hospital cardiologists at patients’ first visit and subsequently as appropriate using General Electric, Model Vivid E9, Horten, Norway with M5S probe or General Electric, Model S6, Horten, Norway with M4S probe. Measurements were taken in accordance with recommendations of the American Society of Echocardiography and the British Society of Echocardiography [13,14].

### Blood pressure measurement

Blood pressure check using appropriate upper-arm cuff sizes was done by physicians with a mercury sphygmomanometer (Accosson, London, UK) after 3–5 minutes of rest in a sitting position. BP recheck was done after a minute, and then recorded down as office BP. SBP and DBP were measured using Korotkoff sounds I at the first appearance of the heart sounds and V at the disappearance of the heart sounds respectively. All patients were counselled on lifestyle modification, adherence, and prescriptions were reviewed by the pharmacy department for medications that increase BP such as nonsteroidal anti-inflammatories, antacids and sodium-containing proton pump inhibitors, sympathomimetic agents, steroids, contraceptive pills, antidepressants, and immunosuppressants.

All patients were encouraged to get the Omron M2 model BP monitor, which underwent calibration in the clinic, for upper-arm home BP monitoring (HBPM). They were taught by the physicians to measure their BP after sitting comfortably for at least five minutes. They were to take three BP readings, one minute apart, in the morning (after urinating) and at bedtime. The last two BP readings were recorded in their BP diary. The patients were asked to measure their BP at least three times a week and take daily measurements one week before clinic visit. The average of the last two BP readings of all daily measurements one week before clinic visit was recorded as home BP.

### Adherence and follow-up

Adherence was determined using prescription and pill count monitoring. Prescriptions were monitored for processing and dispensing by the hospital pharmacy, and patients brought their medications to the clinic for sighting and pill count. Patients were seen at two to four weeks following initial visit, and then monthly until BP control. Doses of medications were titrated upwards at each visit until optimal or best-tolerated doses were achieved.

### Definitions

- Hypertension was diagnosed by persistent elevation of office BP ≥ 140/90 mmHg [15], use of antihypertensive medication or prior diagnosis by a physician before referral to our clinic.
- RH was defined as BP ≥ 140/90 mmHg despite the use of ≥ 3 guideline-recommended antihypertensive medications including a diuretic (thiazide/thiazide-like), a renin-angiotensin system (RAS) blocker (angiotensin-converting enzyme inhibitor [ACEI] or angiotensin receptor blocker [ARB]), and a long-acting calcium channel blocker (CCB) at optimal or best- tolerated doses [15,16]. RH also included patients on ≥ 4 antihypertensive medications with a BP of < 140/90 mmHg [16]. RH was confirmed with the use of an average home BP of ≤ 135/85 mmHg in the week preceding clinic visit. Patients must have been on antihypertensive medications for more than one month after the first clinic visit. Those with uncontrolled office BP ≥ 140/90 who were not adherent to lifestyle and pharmacological treatment, or not on guideline-recommended anti-hypertensive drugs or with white-coat hypertension were not considered to have RH.
- Pseudo-RH refers to falsely diagnosed RH as a result of inaccurate BP measurement, non-use of guideline-recommended antihypertensive drug combinations, medication non-adherence, white coat hypertension, or treatment inertia [10,16].
- White coat hypertension refers to office BP elevations in the presence of controlled home or ambulatory BP levels [10,16].
- Masked hypertension refers to normal office BP levels in the presence of elevated home or ambulatory BP [10,16].
- Treatment inertia refers to the use of inadequate doses or non-use of guideline-recommended BP-lowering drug combinations [10,16].
- Diabetes mellitus (DM) was diagnosed by fasting blood glucose (FBG) of 7.0 mmol/l or 2-hour plasma glucose level of 11.1 mmol/l [17], and obesity was diagnosed by a body mass index of ≥ 30 kg/m^2^ [18]. Dyslipidemia was diagnosed when any change in lipids (total cholesterol [TC] ≥ 5.2 mmol/l, HDL < 1.0 mmol/l in males and < 1.2 mmol/l in females, LDL ≥ 3.4 mmol/l, and triglycerides [TG] ≥ 1.7 mmol/l), whether isolated or combined, was present [19,20].
- Cardiovascular comorbidities present at first visit or in the course of follow-up were recorded as follows: HF (HF with reduced ejection fraction [HFrEF], HF with mildly reduced ejection fraction [HFmREF] and HF with preserved ejection fraction [HFpEF]), CAD, CVD, CKD, peripheral arterial disease (PAD) and arrhythmias. They were diagnosed by physicians with appropriate tests in line with guideline recommendations [21,22,23,24,25,26,27].
- Alcohol use was defined as current intake of alcohol or cessation of intake of less than one-year duration.
- Tobacco use was defined as current, passive, or past use of tobacco and tobacco products.

### Data Analysis

Categorical variables are expressed as proportions and percentages while continuous variables are expressed as means ± standard deviation or as ranges. The association of variables of all patients with the presence or absence of RH was tested by chi-squared test and independent T-test where appropriate. Fisher’s exact test was used for categorical data with an expected cell size of less than 5. Multivariable logistic regression with model-fitting statistics was used to draw an association between statistically significant predictor and outcome variables from univariable models. P- value < 0.05 is taken as statistically significant. Data was analyzed using the SPSS version 26 software for Windows.

## Results

Table 1 shows the baseline characteristics of participants by RH. The mean age was 55.6±12.9 years and there were more females (56.1%) than males (43.9%). More males however had RH (51.4%). Participants with RH had a significantly longer duration of hypertension compared with those without (p < 0.001). At the univariate level, CV risk factors such as DM (p = 0.018), obesity (p < 0.001), and family history of hypertension (p = 0.011) showed significant association with RH. Fasting blood glucose (p = 0.010) and serum creatinine (p = 0.009) were significantly higher, and eGFR was significantly lower (p = 0.018) respectively in those with RH compared with those without RH. Also, office BP at the first visit and control in those with RH were significantly higher than in those without RH (p < 0.001). The frequency of controlled RH, uncontrolled RH and combined RH were 12.3%, 3.3%, (p < 0.001) and 15.5% respectively while the frequency of white coat hypertension was 1.7% (p = 0.073).

**Table 1:** Baseline characteristics of participants by resistant hypertension.


	TOTAL n = 1378	Non-RH (n = 1164)	RH (n = 214)	P-VALUE

Age (years)	55.6 ± 12.9	55.6 ± 13.1	55.5 ± 11.8	0.990

Sex				

Male	606 (43.9%)	496 (42.6%)	110 (51.4%)	0.017

Female	773 (56.1%)	669 (57.4%)	104 (48.6%)	

Duration of hypertension (months)	98.6 ± 99.0	92.5 ± 95.9	129.1 ± 108.7	<0.001

Fasting blood glucose (mmol/l)	6.0 ± 2.4	6.0 ± 2.2	6.5 ± 3.1	0.010

Diabetes Mellitus	284 (20.6%)	227 (19.5%)	57 (26.6%)	0.018

Family history of hypertension	783 (56.7%)	651 (55.9%)	132 (61.7%)	0.011

Alcohol use	415 (30.1%)	341 (29.3%)	74 (34.6%)	0.198

Tobacco use	79 (5.7%)	62 (5.3%)	17 (7.9%)	0.204

First office SBP (mmHg)	145 ± 23	143 ± 21	160 ± 23	<0.001

First office DBP (mmHg)	88 ± 14	87 ± 14	93 ± 15	<0.001

Office SBP at control (mmHg)	125 ± 14	123 ± 13	132 ± 17	<0.001

Office DBP at control (mmHg)	77 ± 9	77 ± 9	79 ± 12	<0.001

Total cholesterol (mmol/l)	5.1 ± 1.2	5.1 ± 1.2	5.1 ± 1.4	0.542

High-density lipoprotein (mmol/l)	1.6 ± 0.6	1.6 ± 0.6	1.6 ± 0.6	0.525

Low-density lipoprotein (mmol/l)	3.0 ± 1.1	3.0 ± 1.1	3.1 ± 1.2	0.616

Triglyceride (mmol/l)	1.2 ± 0.6	1.2 ± 0.6	1.3 ± 0.7	0.050

Dyslipidemia	917 (70.6%)	770 (70.5%)	147 (71.4%)	0.807

Obesity (BMI > 30 kg/m2)	660 (47.9%)	535 (45.9%)	125 (59.0%)	<0.001

Number of antihypertensives	2.6 ± 1.1	2.4 ± 1.0	4.1 ± 0.7	<0.001

No of office visits before BP control	2.87 ± 1.58	2.66 ± 1.20	3.96 ± 2.59	<0.001

BP Control				<0.001

Controlled Non-RH	1105 (80.1%)	1105 (94.8%)	0.0 (0%)	

Controlled RH	169 (12.3%)	0.0 (0%)	169 (79.0%)	

Uncontrolled Non-RH	60 (4.4%)	60 (5.2%)	0.0 (0%)	

Uncontrolled RH	45 (3.3%)	0.0 (0.0%)	45 (21.0%)	

White coat hypertension	23 (1.7%)	16 (1.4%)	7 (3.3%)*	0.073

Serum creatinine (µmol/l)	98.0 ± 36.8	96.9 ± 37.4	103.6 ± 33.1	0.009

eGFR (mls/min/1.73 m^2^)	72.3 ± 19.8	72.9 ± 19.8	69.4 ± 19.5	0.018

LVMI (g/m^2^)	107.9 ± 36.2	107.4 ± 36.7	111.0 ± 33.1	0.177

Ejection fraction (%)	64 ± 14	64 ± 15	67 ± 12	0.004

Heart Failure	207 (15.0%)	188 (16.1%)	19 (8.9%)	0.006

HFrEF/HFmrEF	145 (10.5%)	137 (11.8%)	8 (3.7%)	0.002

HFpEF	62 (4.5%)	51 (4.4%)	11 (5.1%)	0.002

Cerebrovascular Disease	86 (6.2%)	63 (5.4%)	23 (10.7%)	0.003

Chronic Kidney Disease	35 (2.5%)	25 (2.1%)	10 (4.7%)	0.031

Coronary Artery Disease	27 (2.0%)	22 (1.9%)	5 (2.3%)	0.664


BP- Blood pressure, BMI- Body mass index, DBP- Diastolic blood pressure, eGFR- Estimated glomerular filtration rate, HFpEF- Heart failure with preserved ejection fraction, HFmrEF- Heart failure with mildly reduced ejection fraction, HFrEF- Heart failure with reduced ejection fraction, LVMI- Left ventricular mass index, RH- Resistant hypertension, No- Number, SBP- Systolic blood pressure.

The number of antihypertensive medications taken was significantly higher in RH participants (p < 0.001), and ejection fraction (p = 0.004), HF (p = 0.006), HFrEF/HFmrEF (p = 0.002), HFpEF (p = 0.002), CVD (p = 0.003), and CKD (p = 0.031) were significantly higher in those with RH. Patients with RH visited the clinic more times before BP control than non-RH patients (p < 0.001). However, TC, HDL, LDL, TG, alcohol and tobacco use, dyslipidemia, left ventricular mass index, and CAD were not significantly associated with RH (p > 0.05).

In Table 2, the multivariable model showed a significant and positive association of male sex (p = 0.002), obesity (p < 0.001), DM (p = 0.032), CVD (p = 0.011), CKD (p = 0.008) and HFpEF (p = 0.017) with RH. Family history of hypertension (p = 0.053) did not show a significant association while those with HF (p < 0.001) were less likely to have RH compared with those without RH.

**Table 2:** Multivariable logistic regression analysis of risk factors and cardiovascular comorbidities associated with resistant hypertension.


OUTCOME (RESISTANT HYPERTENSION)		

CO-VARIATES	ADJUSTED ODDS RATIO (95% CI)	P-VALUE

Sex (male)	1.62 (1.19–2.21)	0.002

Family history of hypertension	1.40 (0.99–1.96)	0.053

Obesity	1.81 (1.32–2.47)	<0.001

Diabetes Mellitus	1.46 (1.03–2.06)	0.032

HFpEF	3.36 (1.25–9.07)	0.017

HF	0.26 (0.12–0.55)	<0.001

Cerebrovascular disease	2.00 (1.17–3.41)	0.011

Chronic kidney disease	3.01 (1.33–6.84)	0.008


HF- Heart failure, HFpEF- Heart failure with preserved ejection fraction, Model fit p < 0.001, Cox & Snell R Square of 0.044, Nagelkerke R Square of 0.076, Hosmer and Lemeshow Test of 0.935.

Table 3 shows the medications and medication combinations used by the participants. Patients with RH were on guideline recommended combinations of thiazide or thiazide-like diuretics (100%), CCB (100%), and RAS blockers (100%) [ARBs (57.5%) and ACE inhibitors (42.5%)]. 66.4% of the patients with RH were on beta-blockers while 19.6% and 15.4% were on alpha-blockers and mineralocorticoid antagonists (spironolactone/eplerenone) respectively. For all the hypertensives, free medication combinations were used in 57.4% of cases, 6.4% as single pill combinations (SPCs) and 20.2% were mixed (free and SPCs) [p < 0.001].

**Table 3:** Medications and their combinations used by the participants.


PARAMETERS	TOTAL (n = 1378)	Non-RH (n = 1164)	RH (n = 214)	P-VALUE

**Medications**				

Calcium channel blockers	927 (67.2%)	713 (61.2%)	214 (100%)	<0.001

Diuretics (Thiazides, Thiazide-like and Loop) **	834 (60.5	620 (53.2%)	214 (100%)	<0.001

Mineralocorticoid antagonists (Spironolactone/Eplerenone)	104 (7.5%)	71 (6.1%)	33 (15.4%)	<0.001

ACE Inhibitors	540 (39.2%)	449 (38.5%)	91 (42.5%)	0.273

Angiotensin Receptor Blockers	562 (40.8%)	439 (37.7%)	123 (57.5%)	<0.001

Beta-blockers	502 (36.4%)	360 (30.9%)	142 (66.4%)	<0.001

Alpha-blockers	55 (4.0%)	13 (1.1%)	42 (19.6%)	<0.001

Centrally acting (Alpha Methyl Dopa)	50 (3.6%)	44 (3.8%)	6 (2.8%)	0.484

Hydralazine	5 (0.4%)	2 (0.2%)	3 (1.4%)	0.006*

**Medication combinations**				

Free	791 (57.4%)	696 (59.7%)	95 (44.4%)	<0.001

Single pill combination	88 (6.4%)	86 (7.4%)	2 (0.9%)	<0.001

Mixed	279 (20.2%)	162 (13.9%)	117 (54.7%)	<0.001


*Fisher’s exact test, **Loop diuretics not used in those with RH. ACE- Angiotensin-converting enzyme. Mixed- use of free and single pill combinations together, RH- Resistant hypertension.

## Discussion

The prevalence of combined RH in this study is 15.5%, with 3.3% for uncontrolled RH and 12.3% for controlled RH. To our knowledge, this is the first Nigerian and African RH study to use guideline-recommended antihypertensive combinations wholly, and the first to examine the association of RH with CV comorbidities.

Previous African studies on RH revealed that the prevalence of RH range from 4.9 to 19% [28,29,30,31,32,33,34,35]. The wide range reflects the different definitions that were used for the different studies, partly because of the evolving understanding of RH in the last two decades. In a systematic review and meta-analysis, Nansseu et al. noted that the patients used in the five studies included could be classified as true RH, controlled RH, or pseudo-RH depending on the definition used [28]. In the only study in the review that measured out-of-office BP, Yameogo et al. found a prevalence of 14.6% among 692 hypertensives with office BP of ≥ 140/90 mmHg and ambulatory BP of ≥ 130/80 mmHg, but without the use of guideline-recommended antihypertensive combinations [31]. More recently, Kuntonda et al., in a retrospective cohort study, reported a prevalence of 9.4% for true RH after 24-hour ABPM [29], while in another recent Ghanaian multi-center study, the prevalence of apparent RH was 18.9% [30]. Our study’s prevalence is within the range of previous studies but may approximate the true prevalence because of the use of guideline-recommended antihypertensives, longer duration of hypertension, longitudinal BP control through optimal or best tolerated antihypertensive doses, and assessment of adherence and lifestyle modification. Previous studies conducted in African populations also did not make a distinction between uncontrolled and controlled RH. Globally, the prevalence of RH in treated hypertensives varies widely from 1.9 to 30% [10,15] depending on the population studied and the methodology used. Using the definition of the European Society of Cardiology (ESC), it has been suggested that the true prevalence of RH is likely to be < 10% among treated patients [10,15]. Therefore, using the same ESC definition, our study suggests that the prevalence of uncontrolled RH in a treated hypertensive Nigerian population may be less than 5%. However, studies have shown that both controlled and uncontrolled RH confer morbidity and mortality in hypertensive patients [3,36]. In a cross-sectional study of 470,386 hypertensive individuals carried out by Sim et al., the risk of end-stage renal disease (ESRD), ischemic heart disease, congestive heart failure, cerebrovascular accident, and mortality was higher in those with controlled and uncontrolled RH compared to those without RH [3]. Sim et al. also reported the prevalence of uncontrolled RH, controlled RH, and combined RH to be 7.9%, 4.9%, and 12.8% respectively [36]. The prevalence of controlled and combined RH is higher in our study, and this may be the result of higher baseline office BP of 160/93 (23/15) mmHg in our study, compared to 143/74 (20/13) mmHg in their study. This is not surprising because of the lower levels of awareness, treatment, and control of hypertension in African populations compared to non-African populations [37]. The prevalence of uncontrolled RH is higher in the study by Sim et al. compared to ours. It is noteworthy that Sim and colleagues did not confirm RH with out-of-office BP measurements, hence the true prevalence is likely to be lower. Though the prevalence of uncontrolled RH is lower compared to controlled RH in this Nigerian cohort, both groups are at an increased risk of morbidity and mortality.

Risk factors that have been associated with RH in African and non-African populations include increasing age, obesity, DM, family history of hypertension, dyslipidemia, metabolic syndrome, and sedentary living [1,2,3,4,5,6,7,29,30,31,32,33,34,35]. In our study, we found that hypertensive patients who are male, obese, and diabetic are more likely to have RH. With increasing rates of these risk factors of RH in Nigeria and Africa, this suggests that the burden of RH will continue to rise with a substantial increase in CV morbidity and mortality. Therefore, strategies geared toward preventing and treating these risk factors of RH need to be reinforced.

After multivariable adjustment, our study showed that CVD and CKD are two and three times, respectively, more likely to be present in hypertensives with RH. This is similar to greater risks in RH compared with non-RH patients in a large ethnically diverse hypertensive population [3,36]. Data from longitudinal, retrospective, registry, and survey analyses, show that RH is not only associated with increased CV events and all-cause mortality but also with CHF [1,2,3,4,5,6,7]. Our study showed that compared with those without HF, patients with HF were less likely to have RH while those with HFpEF were 3 times more likely to have RH than those with other forms of HF. The higher number of patients in this hypertensive cohort with HFrEF and HFmrEF may account for the difference between HF and HFpEF. The protective effect of HFrEF has been previously shown by Jin et al in their study of 1,288 patients admitted for HF in China [38]. Our study showed for the first time in a Nigerian population that CV comorbidities are more prevalent in hypertensives with RH. This increased risk of CV morbidities in RH populations implies a higher healthcare and economic burden. Hence, concerted and collaborative action from the primary care level to ensure early identification of RH, and prompt referral for specialist care are imperative to reduce further morbidity and mortality.

The prevalence of white coat hypertension in our study is low at 1.7%. A low prevalence of white coat hypertension is expected when office BP is based on repeated measurements as seen in this study [15]. All the patients with RH in our study had guideline-recommended antihypertensive combinations. This is the first to our knowledge in Nigeria and Africa, eliminating a common cause of pseudo-RH in treatment inertia. Our study showed that SPC use (6.4%) was the lowest of the medication combinations. SPCs are expensive, and our patients mostly paid out of pocket for their medications. Medication adherence may therefore be jeopardized in patients not well motivated to take free pills, leading to poor BP control. The use of cheaper generic SPCs may enhance adherence and BP control consequently. In addition, the incorporation of fourth-line medications such as mineralocorticoid antagonists, beta blockers, and doxazosin in various SPCs for RH may greatly enhance adherence. These would lead to a reduction in the morbidity and mortality associated with RH.

One limitation of our study is that we were not able to confirm adherence with urinary or serum metabolite testing. We used monitoring of prescription orders and pill count to assess adherence. As much as 53% of patients were reported to be non-adherent by Jung et al. when urinary drug or drug metabolite levels were measured in patients referred for uncontrolled RH [39]. However, biochemical assays are expensive and are not readily available [40]. We used HBPM to confirm RH in our study instead of ABPM because of the lack of health insurance to cover the cost of ABPM for most of our patients. Guidelines recommend ABPM and/or HBPM to confirm RH [15,16,41,42]. Our data on CV comorbidities should be interpreted with caution because it combined events that were present at recruitment and events that occurred at follow-up. However, our relatively large sample size, use of recommended guideline medication combinations, HBPM to confirm RH, and assessment of CV morbidities for the first time in African patients are strengths of this study. Further studies are needed in Africa to prospectively ascertain the burden of mortality of RH.

## Conclusion

This study showed the prevalence of RH including uncontrolled and controlled in a treated hypertensive Nigerian cohort. This is likely a true representation of RH in Nigerian hypertensives given that reatment inertia and pseudo-RH were excluded. Patients with RH were more likely to be male, obese, and diabetic. For the first time in a Nigerian and African cohort, our study demonstrated the association of RH with CVD, CKD, and HFpEF. Our findings highlight the heavy burden of RH which calls for early diagnosis of RH to reduce morbidity and mortality.

## Data Accessibility Statement

The datasets used and/or analyzed during the current study are available from the corresponding author on request.

## References

[1] Egan BM, Kai B, Wagner CS, Henderson JH, Chandler AH, Sinopoli A. Blood pressure control provides less cardiovascular protection in adults with than without apparent treatment-resistant hypertension. J Clin Hypertens (Greenwich). 2016; 18: 817–824. DOI: 10.1111/jch.12773 26856795 PMC5837039

[2] Thomas G, Xie D, Chen HY, Anderson AH, Appel LJ, Bodana S, et al., CRIC Study Investigators. Prevalence and prognostic significance of apparent treatment-resistant hypertension in chronic kidney disease: report from the Chronic Renal Insufficiency Cohort Study. Hypertension. 2016; 67: 387–396. DOI: 10.1161/HYPERTENSIONAHA.115.06487 26711738 PMC4713320

[3] Sim JJ, Bhandari SK, Shi J, Reynolds K, Calhoun DA, Kalantar-Zadeh K, et al. Comparative risk of renal, cardiovascular, and mortality outcomes in controlled, uncontrolled resistant, and nonresistant hypertension. Kidney Int. 2015; 88: 622–632. DOI: 10.1038/ki.2015.142 25945406 PMC4556588

[4] Irvin MR, Booth JN 3rd, Shimbo D, Lackland DT, Oparil S, Howard G, et al. Apparent treatment-resistant hypertension and risk for stroke, coronary heart disease, and all-cause mortality. J Am Soc Hypertens. 2014; 8: 405–413. DOI: 10.1016/j.jash.2014.03.003 24952653 PMC4120268

[5] Smith SM, Gong Y, Handberg E, Messerli FH, Bakris GL, Ahmed A, et al. Predictors and outcomes of resistant hypertension among patients with coronary artery disease and hypertension. J Hypertens. 2014; 32: 635–643. DOI: 10.1097/HJH.0000000000000051 24299915 PMC4118668

[6] Smith SM, Huo T, Delia Johnson B, Bittner V, Kelsey SF, Vido Thompson D, et al. Cardiovascular and mortality risk of apparent resistant hypertension in women with suspected myocardial ischemia: a report from the NHLBI-sponsored WISE Study. J Am Heart Assoc. 2014; 3: e000660. DOI: 10.1161/JAHA.113.000660 24584740 PMC3959684

[7] Daugherty SL, Powers JD, Magid DJ, Tavel HM, Masoudi FA, Margolis KL, et al. Incidence and prognosis of resistant hypertension in hypertensive patients. Circulation. 2012; 125: 1635–1642. DOI: 10.1161/CIRCULATIONAHA.111.068064 22379110 PMC3343635

[8] Mansia G, De Backer G, Dominiczak A, Cifkova R, Fagard R, Germano G, et al. 2007 ESH-ESC Guidelines for the management of arterial hypertension: The task force for the management of arterial hypertension of the European Society of Hypertension (ESH) and of the European Society of Cardiology (ESC). Blood Press. 2007; 16(3): 135–232. DOI: 10.1080/08037050701461084 17846925

[9] Calhoun DA, Jones D, Textor S, Goff DC, Murphy TP, Toto RD, et al. Resistant hypertension: diagnosis, evaluation, and treatment. A scientific statement from the American Heart Association Professional Education Committee of the Council for High Blood Pressure Research. Hypertens Dallas Tex. 1979. 2008; 51(6): 1403–1409. DOI: 10.1161/HYPERTENSIONAHA.108.189141 18391085

[10] Williams B, Mancia G, Spiering W, Agabiti Rosei E, Azizi M, Burnier M, et al. 2018 Practice Guidelines for the management of arterial hypertension of the European Society of Cardiology and the European Society of Hypertension. Blood Press. 2018 Dec; 27(6): 314–340. Erratum in: Blood Press. 2019 Feb; 28(1): 74. PMID: 30380928. DOI: 10.1080/08037051.2018.1527177 30380928

[11] Inker LA, Eneanya ND, Coresh J, Tighiouart H, Wang D, Sang Y, et al. Chronic kidney disease epidemiology collaboration. New creatinine- and cystatin C-based equations to estimate GFR without race. N Engl J Med. 2021; 385(19): 1737–1749. DOI: 10.1056/NEJMoa2102953 34554658 PMC8822996

[12] Kligfield P, Gettes L, Bailey JJ, Childers R, Deal BJ, Hancock EW, et al. Recommendations for the standardization and interpretation of the electrocardiogram: Part 1: The electrocardiogram and its technology: A scientific statement from the American Heart Association Electrocardiography and Arrhythmias Committee, Council on Clinical Cardiology; the American College of Cardiology Foundation; and the Heart Rhythm Society: Endorsed by the International Society for Computerized Electrocardiology. Circulation. 2007; 49: 1109–112. DOI: 10.1016/j.jacc.2007.01.024 17349896

[13] Lang RM, Bierig M, Devereux RB, Flachskampf FA, Foster E, Pellikka PA, et al. Chamber Quantification Writing Group; American Society of Echocardiography’s Guidelines and Standards Committee. J Am Soc Echocardiography. 2005; 18: 1440–1463. DOI: 10.1016/j.echo.2005.10.005 16376782

[14] Harkness A, Ring L, Augustine DX, Oxborough D, Robinson S, Sharma V; Education Committee of the British Society of Echocardiography. Normal reference intervals for cardiac dimensions and function for use in echocardiographic practice: A guideline from the British Society of Echocardiography. Echo Res Pract. 2020; 7(1): G1–G18. Erratum in: Echo Res Pract. 2020; 7(1): X1. DOI: 10.1530/ERP-19-0050 32105051 PMC7040881

[15] Mancia G, Kreutz R, Brunström M, Burnier M, Grassi G, Januszewicz A, et al., Authors/Task Force Members. 2023 ESH Guidelines for the management of arterial hypertension. The Task Force for the management of arterial hypertension of the European Society of Hypertension Endorsed by the European Renal Association (ERA) and the International Society of Hypertension (ISH). J Hypertens. 2023 Jun 21. DOI: 10.1097/HJH.0000000000003480 37345492

[16] Carey RM, Calhoun DA, Bakris GL, Brook RD, Daugherty SL, Dennison-Himmelfarb CR, et al., American Heart Association Professional/Public Education and Publications Committee of the Council on Hypertension, Council on Cardiovascular and Stroke Nursing, Council on Clinical Cardiology, Council on Genomic and Precision Medicine, Council on Peripheral Vascular Disease, Council on Quality of Care and Outcomes Research, Stroke Council. Resistant hypertension: Detection, evaluation, and management: A scientific statement from the American Heart Association. Hypertension. 2018; 72(5): E53–E90. DOI: 10.1161/HYP.0000000000000084 30354828 PMC6530990

[17] Standards of Medical Care in Diabetes-2016: Summary of Revisions. Diabetes Care. 2016; 39(Suppl 1): S4–S5. DOI: 10.2337/dc16-S003 26696680

[18] Jensen MD, Ryan DH, Apovian CM, Ard JD, Comuzzie AG, Donato KA, et al., American College of Cardiology/American Heart Association Task Force on Practice Guidelines, Obesity Society. 2013 AHA/ACC/TOS guideline for the management of overweight and obesity in adults: A report of the American College of Cardiology/American Heart Association Task Force on Practice Guidelines and The Obesity Society. Circulation. 2014; 129(25 Suppl 2): S102–S138. Erratum in: Circulation. 2014; 129(25 Suppl 2): S139–S140. DOI: 10.1161/01.cir.0000437739.71477.ee 24222017 PMC5819889

[19] NCEP, ATP III Executive Summary National Cholesterol Education Program National Heart, Lung, and Blood Institute National Institutes of Health NIH Publication. 2001; No. 01–3670.

[20] Bauman CD, Bauman JM, Mourão DM, Pinho L, Brito MFSF, Carneiro ALG, et al. Dyslipidemia prevalence in adolescents in public schools. Rev Bras Enferm. 2020; 73(3): e20180523. DOI: 10.1590/0034-7167-2018-0523 32321121

[21] Kirchhof P, Benussi S, Kotecha D, Ahlsson A, Atar D, Casadei B, et al., ESC Scientific Document Group. 2016 ESC Guidelines for the management of atrial fibrillation developed in collaboration with EACTS. Eur Heart J. 2016; 37(38): 2893–2962. DOI: 10.1093/eurheartj/ehw210 27567408

[22] Ponikowski P, Voors AA, Anker SD, Bueno H, Cleland JG, Coats AJ, et al., Authors/Task Force Members; Document Reviewers. 2016 ESC guidelines for the diagnosis and treatment of acute and chronic heart failure: The task force for the diagnosis and treatment of acute and chronic heart failure of the European Society of Cardiology (ESC). Developed with the special contribution of the Heart Failure Association (HFA) of the ESC. Eur J Heart Fail. 2016; 18(8): 891–975. DOI: 10.1002/ejhf.592 27207191

[23] Amsterdam EA, Wenger NK, Brindis RG, Casey DE Jr., Ganiats TG, Holmes DR Jr., et al., ACC/AHA Task Force Members. 2014 AHA/ACC guideline for the management of patients with non-ST-elevation acute coronary syndromes: A report of the American College of Cardiology/American Heart Association Task Force on Practice Guidelines. Circulation. 2014; 130(25): e344–426. Erratum in: Circulation. 2014; 130(25): e433–434. DOI: 10.1161/CIR.0000000000000134 25249585

[24] Levine GN, Bates ER, Blankenship JC, Bailey SR, Bittl JA, Cercek B, et al. 2015 ACC/AHA/SCAI focused update on primary percutaneous coronary intervention for patients with ST-elevation myocardial Infarction: An update of the 2011 ACCF/AHA/SCAI guideline for percutaneous coronary intervention and the 2013 ACCF/AHA guideline for the management of ST-elevation myocardial infarction: A report of the American College of Cardiology/American Heart Association Task Force on Clinical Practice Guidelines and the Society for Cardiovascular Angiography and Interventions. Catheter Cardiovasc Interv. 2016; 87(6): 1001–1019. DOI: 10.1002/ccd.26325 26489034

[25] Fihn SD, Blankenship JC, Alexander KP, Bittl JA, Byrne JG, Fletcher BJ, et al. 2014 ACC/AHA/AATS/PCNA/SCAI/STS focused update of the guideline for the diagnosis and management of patients with stable ischemic heart disease: A report of the American College of Cardiology/American Heart Association Task Force on Practice Guidelines, and the American Association for Thoracic Surgery, Preventive Cardiovascular Nurses Association, Society for Cardiovascular Angiography and Interventions, and Society of Thoracic Surgeons. Circulation. 2014; 130(19): 1749–1767. DOI: 10.1161/CIR.0000000000000095 25070666

[26] Jauch EC, Saver JL, Adams HP Jr., Bruno A, Connors JJ, Demaerschalk BM, et al., American Heart Association Stroke Council; Council on Cardiovascular Nursing; Council on Peripheral Vascular Disease; Council on Clinical Cardiology. Guidelines for the early management of patients with acute ischemic stroke: A guideline for healthcare professionals from the American Heart Association/American Stroke Association. Stroke. 2013; 44(3): 870–947. DOI: 10.1161/STR.0b013e318284056a 23370205

[27] Hemphill JC 3rd, Greenberg SM, Anderson CS, Becker K, Bendok BR, Cushman M, et al., American Heart Association Stroke Council, Council on Cardiovascular and Stroke Nursing, Council on Clinical Cardiology. Guidelines for the management of spontaneous intracerebral hemorrhage: A guideline for healthcare professionals from the American Heart Association/American Stroke Association. Stroke. 2015; 46(7): 2032–2060. DOI: 10.1161/STR.0000000000000069 26022637

[28] Nansseu JR, Noubiap JJ, Mengnjo MK, Aminde LN, Essouma M, Jingi AM, et al. The highly neglected burden of resistant hypertension in Africa: A systematic review and meta-analysis. BMJ Open. 2016; 6(9): e011452. DOI: 10.1136/bmjopen-2016-011452 PMC5051381 27650760

[29] Kuntonda DK, Lepira FB, Lubenga Y, Makulo JRR, Nkodila A, Otshudi N, et al. True resistant hypertension among treated hypertensive black patients. A clinical-based cross-sectional study. World Journal of Cardiovascular Diseases. 2020; 10: 278–293. DOI: 10.4236/wjcd.2020.105026

[30] Ayisi-Boateng NK, Mohammed A, Opoku DA, Sarfo FS. Frequency & factors associated with apparent resistant hypertension among Ghanaians in a multicenter study. J Clin Hypertens (Greenwich). 2020; 22(9): 1594–1602. DOI: 10.1111/jch.13974 32815641 PMC8029809

[31] Yaméogo NV, Samadoulougou AK, Kagambèga LJ, Millogo GR, Yaméogo AA, Kologo KJ, et al. Epidemiological characteristics and clinical features of black African subject’s resistant hypertension. Ann Cardiol Angeiol (Paris). 2014; 63: 83–88. DOI: 10.1016/j.ancard.2014.01.002 24492012

[32] Youmbissi TJ, Meli J, Kinkela MN, Ngu JL. Resistant hypertension in Yaounde. West Afr J Med. 1994; 13: 175–178. 7841110

[33] Salako BL, Ayodele OE. Observed factors responsible for resistant hypertension in a teaching hospital setting. Afr J Med Med Sci. 2003; 32: 151–154. 15032461

[34] Thinyane KH, Mothebe T, Sooro M, Namole LD, Cooper V. An observational study of hypertension treatment and patient outcomes in a primary care setting. Pan Afr Med J. 2015; 20: 424. DOI: 10.11604/pamj.2015.20.424.5040 26309457 PMC4537907

[35] Bachir Cherif A, Taleb A, Temmar M, et al. Prevalence and causes of resistant hypertension in specialized consultation in the area of Blida (Algeria). J Hypertens. 2015; 33(e-Supplement 1): e498. DOI: 10.1097/01.hjh.0000468959.33385.5e

[36] Sim JJ, Bhandari SK, Shi J, Liu IL, Calhoun DA, McGlynn EA, et al. Characteristics of resistant hypertension in a large, ethnically diverse hypertension population of an integrated health system. Mayo Clin Proc. 2013; 88: 1099–1107. DOI: 10.1016/j.mayocp.2013.06.017 24079679 PMC3909733

[37] Kadiri S, Arogundade F, Arije A, Omotoso A, Onwubere B, Aderibigbe A, et al. Guidelines for the Management of Hypertension in Nigeria 2020: A Revised Recommendation for Health Care Providers from the Nigerian Hypertension Society. Tropical Journal of Nephrology. 2020; 15: 1.

[38] Jin CN, Liu M, Sun JP, Fang F, Wen YN, Yu CM, et al. The prevalence and prognosis of resistant hypertension in patients with heart failure. PLoS One. 2014; 9(12): e114958. DOI: 10.1371/journal.pone.0114958 25490405 PMC4260939

[39] Jung O, Gechter JL, Wunder C, Paulke A, Bartel C, Geiger H, et al. Resistant hypertension? Assessment of adherence by toxicological urine analysis. J Hypertens. 2013; 31: 766–774. DOI: 10.1097/HJH.0b013e32835e2286 23337469

[40] Hameed MA, Dasgupta I. Medication adherence and treatment-resistant hypertension: a review. Drugs Context. 2019 Feb 4; 8: 212560. DOI: 10.7573/dic.212560 30774692 PMC6365088

[41] Stergiou GS, Palatini P, Parati G, O’Brien E, Januszewicz A, Lurbe E, et al. European Society of Hypertension Council and the European Society of Hypertension Working Group on Blood Pressure Monitoring and Cardiovascular Variability. 2021 European Society of Hypertension practice guidelines for office and out-of-office blood pressure measurement. J Hypertens. 2021; 39(7): 1293–1302. DOI: 10.1097/HJH.0000000000002843 33710173

[42] Whelton PK, Carey RM, Mancia G, Kreutz R, Bundy JD, Williams B. Harmonization of the American College of Cardiology/American Heart Association and European Society of Cardiology/European Society of Hypertension Blood Pressure/Hypertension Guidelines: Comparisons, Reflections, and Recommendations. J Am Coll Cardiol. 2022; 80(12): 1192–1201. DOI: 10.1016/j.jacc.2022.07.005 35965201

